# Optimal therapeutic strategy for those with chronic kidney disease and peripheral artery disease receiving peripheral vascular intervention

**DOI:** 10.1002/clc.23483

**Published:** 2020-10-19

**Authors:** Teruhiko Imamura

**Affiliations:** ^1^ Second Department of Internal Medicine University of Toyama Toyama Japan

To the Editor

Narcisse and colleagues demonstrated that patients with chronic kidney disease (CKD) had a greater risk for all‐cause mortality following peripheral vascular intervention (PVI) despite the high procedural success and low amputation rates.^1^ Several concerns have been raised.

The authors stated that PVI did not effectively mitigate the risk of death.^1^ Data on causes of death would more clarify the causality among peripheral artery disease, PVI procedure, and death.

The authors investigated the impact of CKD on clinical outcomes following the PVI procedure.^1^ Another statistical approach might be stratifying patients by a new cutoff of estimated glomerular filtration rate calculated by the receiver operating characteristic analysis.

Finally, the authors stated that aggressive medical therapies with proven mortality benefit should likely be the focus in those with CKD.^1^ It might be interesting to perform a subanalysis only among the CKD group to further investigate significant therapeutic factors associating with postprocedural better clinical outcomes.

## DATA AVAILABILITY STATEMENT

Data sharing is not applicable to this article as no new data were created or analyzed in this study.
